# A posteriori metadata from automated provenance tracking: integration of AiiDA and TCOD

**DOI:** 10.1186/s13321-017-0242-y

**Published:** 2017-11-14

**Authors:** Andrius Merkys, Nicolas Mounet, Andrea Cepellotti, Nicola Marzari, Saulius Gražulis, Giovanni Pizzi

**Affiliations:** 1 Theory and Simulation of Materials (THEOS) and National Centre for Computational Design and Discovery of Novel Materials (MARVEL), 1015 Lausanne, Switzerland; 2 Institute of Biotechnology, Vilnius University, Saulėtekio al. 7, 10257 Vilnius, Lithuania; 3 Faculty of Mathematics and Informatics, Vilnius University, Naugarduko st. 24, 03225 Vilnius, Lithuania

**Keywords:** DFT, Reproducibility, Provenance, Open data, Ontology, Materials science

## Abstract

In order to make results of computational scientific research findable, accessible, interoperable and re-usable, it is necessary to decorate them with standardised metadata. However, there are a number of technical and practical challenges that make this process difficult to achieve in practice. Here the implementation of a protocol is presented to tag crystal structures with their computed properties, without the need of human intervention to curate the data. This protocol leverages the capabilities of AiiDA, an open-source platform to manage and automate scientific computational workflows, and the TCOD, an open-access database storing computed materials properties using a well-defined and exhaustive ontology. Based on these, the complete procedure to deposit computed data in the TCOD database is automated. All relevant metadata are extracted from the full provenance information that AiiDA tracks and stores automatically while managing the calculations. Such a protocol also enables reproducibility of scientific data in the field of computational materials science. As a proof of concept, the AiiDA–TCOD interface is used to deposit 170 theoretical structures together with their computed properties and their full provenance graphs, consisting in over 4600 AiiDA nodes.

## Background

Modelling and simulation are commonly identified as the third paradigm in scientific understanding, complementing theory and experiment. In particular, computational materials science has developed into an essential field due to two main factors. First, in the past years significant advances have been achieved both in the approximations of the theories used to simulate materials from first-principles [1] and in the codes that implement them (many of which are distributed open-source). Second, these computationally-expensive calculations have been made feasible thanks to the exponential increase of computing power predicted by Moore’s law and the corresponding decrease of the price/performance ratio. As a consequence, large number of properties can nowadays be computed for large families of materials. A number of online databases has appeared in the past few years, like the Materials Project [2], OQMD [3] and AFLOWLIB [4]. However, much effort is still needed to consolidate the knowledge from publications, tagging results with suitable metadata under an established ontology, and preserving at the same time the complete provenance of the computed data to enable reproducibility of the results.

Currently, there are several attempts to define an ontology in the field of theoretical material science, like the European Theoretical Spectroscopy Facility (ETSF) [5, 6], NOMAD [7], OPTiMaDe [8] and the Theoretical Crystallography Open Database (TCOD) [9, 10]. The latter was launched with the aim of collecting the results from several kinds of calculations (DFT, post-HF, QM/MM, etc.), into an open-access resource for long-term archival storage. The TCOD adopts the Crystallographic Information Framework (CIF) format [11], a unified format for reporting and storing the results of experimentally-solved crystal structures, which has been widely adopted and used as the *de facto* standard by most crystallographic journals as well as structural databases like, to mention just a few, the Inorganic Crystal Structure Database (ICSD) [12], the Cambridge Structural Database [13], the American Mineralogist Crystal Structure Database [14], and the Crystallography Open Database (COD) [15, 16]. One of the main advantages of the CIF format is the existence of CIF dictionaries, aimed at defining domain-specific ontologies readable both by humans and by machines [17]. Constraints, units of measurement and interrelationships for data values are specified in order to homogenise the data, eliminate ambiguities and allow for automated validation. Furthermore, since CIF data names are uniquely defined, a CIF file may contain properties from more than one dictionary, making it possible to easily extend and complement the file (e.g. for macromolecular crystallography [18], powder diffraction [19], electron density [20] and experimental material properties [21]). Recently, CIF format version 2.0 (CIF 2, [22]) has been developed with even more features for ontology definition. In the TCOD domain-specific dictionaries have been compiled in order to define an ontology for the hosted data (in particular, the cif_dft.dic dictionary for DFT-based properties, and cif_tcod.dic for the generic metadata related to scientific workflows), and automated checks of CIF files against these dictionaries have been implemented.

The definition of an ontology is not the only challenge that materials science faces; another major issue is the preservation of provenance for result replication. In fact, currently most of the scientific publications provide only a subset of all control parameters, numerical inputs and calculation interrelationships needed to exactly reproduce the published results. This problem can be solved by using provenance-tracking frameworks like AiiDA [23, 24], a high-throughput infrastructure that provides a high-level research environment to automate the execution of computations, systematically store inputs and outputs and their relationships in a graph database (tailored to keep track of the full data provenance) and share results.

In this work we present the integration of the TCOD database with AiiDA, using and enhancing the cif_tcod.dic CIF dictionary [25]. Our integration of a calculation automation framework and an ontology-based database allows for the *a posteriori* deposition of simulation results with automatically recorded metadata. Most importantly, the metadata tagging and deposition can be performed at any time after the calculation has been executed thanks to the automatic provenance tracking provided by AiiDA.

In the following, we first describe the provenance model implemented in AiiDA and explain how we map it to a CIF file. We explain how we address and solve the technical issues that arise in the process, e.g. the inclusion of input and output files within the CIF file and their encoding, how software versions can be tracked, and how to report bibliographic references. We then describe the extensions to the TCOD dictionary that we have implemented, and compare the latter with other existing ontologies. The algorithm of the converters to integrate AiiDA and the TCOD is then illustrated. Finally, we discuss the results obtained and deposited in the TCOD using the codes and algorithms implemented in this work.

## Workflow representation

### Provenance model and directed acyclic graphs

**Fig. 1:**
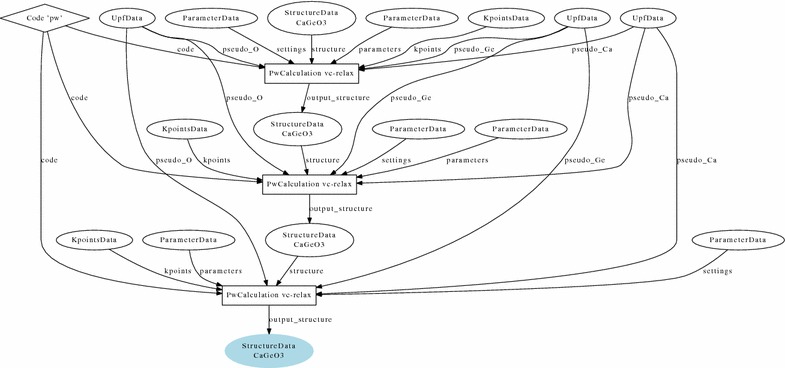
Workflow of TCOD entry 10000008. The workflow consists of three consecutive relaxations of GaGeO$_3$ structure with the *Quantum ESPRESSO* pw.x code. Artifacts (called “data nodes” in AiiDA) are marked as circles and processes (called “calculation nodes” in AiiDA) as rectangles. A special type of AiiDA artifact, a code, is represented by a diamond. Note that in AiiDA the direction of arrows is inverted with respect to the OPM notation [33, 34]

Reproducibility of scientific calculations is a crucial tenet of computational scientific research [26–28], and a system enabling easy replication of exact or modified computations reported in a scientific publication would be an important step in achieving this goal [29]. An important prerequisite is that content (data) must be separated from its presentation (article) [30]. Such separation is implemented for instance in *Sweave* [31], designed specifically for the *R* statistical programming language [32]. In the field of Computational Materials Science, Pizzi et al. have developed AiiDA, a *Python*-based framework for atomistic simulations [24], where data provenance is stored automatically while the simulations and workflows are executed, using a data model elaborating on ideas of the Open Provenance Model (OPM) [33, 34]. OPM suggests to represent whole scientific workflows of data transformations as directed acyclic graphs (DAGs), whose nodes are *artifacts* and vertices are *processes*. An example of DAG of an AiiDA workflow is shown in Fig. 1.

The purpose of this work is to represent faithfully the full provenance of computational workflows, represented by AiiDA in the form of DAGs, within a CIF file. To achieve this goal, we apply the cif_tcod.dic CIF dictionary as follows. We represent workflows by an ordered list of processes (“workflow steps”), so that the execution of such sequence leads to the generation of the workflow results. Workflow steps are represented in a CIF loop using _tcod_computation_* data items, and the sequential numbers of workflow steps are given in _tcod_computation_step. Each step is then defined by its command line string (_tcod_computation_command) and the environment variables (_tcod_computation_environment).

Input and output files and directories (artifacts in the OPM) are described in a CIF loop of _tcod_file_* data items. For the sake of achieving a deterministic order, we recommend providing files and directories sorted by lexicographical order of their full path, with directories always preceding their contents. Moreover, all paths should be relative to the same location throughout all calculations. In particular, the data item _tcod_file_name is used for names of files and directories, whereas the file content is stored in the _tcod_file_contents data item (to comply with the requirements of the CIF file format, the file contents are encoded as described in “File content inclusion in CIF and encoding” section). Since directories do not have file-like contents, the special CIF value ‘.’ (a dot, meaning “data are inapplicable”) must be provided for them. Contents of standard input, output and error of processes (if any) are placed in separate files and linked to the workflow steps using _tcod_computation_input_file, _tcod_computation_stdout and _tcod_computation_stderr data items, accordingly.

**Fig. 2:**
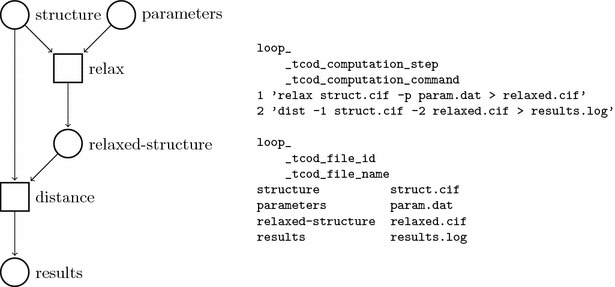
Example of a simple workflow [24]. Graph view (left) and representation in TCOD CIF (right). In the graph view, files are presented as circles and processes as squares. For the sake of brevity and clarity, file contents are not reported here for the TCOD CIF representation

While defining this format, we need to address a technical issue. The CIF format poses restrictions on acceptable CIF values, so that there are cases in which the contents of a file must be encoded (the most obvious case is a file containing a single ‘.’ symbol, that would otherwise be ignored). We have thus developed a few content-encoding protocols that we describe in detail in “File content inclusion in CIF and encoding” section, allowing inclusion of any text and binary file content into CIF text fields. An example of a simple workflow along with its representation in the TCOD CIF format is given in Fig. 2.

### Input data

Initial steps of atomistic simulation workflows usually transform input data (often from external sources or databases) to internal structures. To preserve the full history, it is crucial to maintain a reference to the original data. This is relatively straightforward if the resource is available on the Internet *and* is assigned a permanent URI, DOI or similar resource identifier. Usually, upon retrieval, it is beneficial to supplement such identifier with retrieval date, resource version (if given) and a checksum as a tool for ultimate integrity control.

As part of this work, we have implemented a number of external database importers as part of AiiDA. These provide the user with the possibility to seamlessly import data from several structural databases, including the COD [16], ICSD [12], Material Properties Open Database (MPOD) [21], Open Quantum Materials Database (OQMD) [35], as well as a pseudopotential database, the NNIN/C Pseudopotential Virtual Vault [36]. By means of these importers, the user can both query and fetch data from the respective databases, and then import the relevant entries directly into AiiDA. During this process, we make sure to record information about the source: permanent URIs (where available), versions and checksums. When the AiiDA graph is later exported to CIF format and deposited in the TCOD, the source of the initial structural data is recorded in the cif_tcod.dic data items _tcod_source_structure_* and _tcod_source_database_*.

**Fig. 3:**
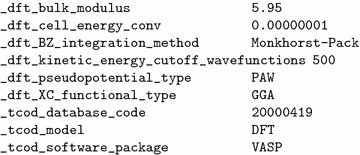
Sample from TCOD entry 20000419. This excerpt displays computational setup and bulk modulus (in GPa), convergence criterion for cell energy and kinetic energy cut-off for wavefunctions (both in eV). Units for each data item are unambiguously defined in the TCOD dictionary

### File content inclusion in CIF and encoding

As described in “Provenance model and directed acyclic graphs” section, in TCOD CIFs we aim at storing an archive of the whole computational workflow used to obtain the properties of a given material within the same TCOD CIF file. As a consequence, the file contents of individual calculation inputs and outputs are stored as values of the _tcod_file_contents data item. However, due to the format restrictions of the CIF format, not all data can be stored unmodified in a CIF 1.1 file text field. In particular, the following restrictions apply:

- the character set is restricted to printable and whitespace ASCII characters, so that CIF files must not contain unescaped binary data and Unicode symbols;
- lines (except the first) must not start with semicolons. This character is reserved as the text field delimiter. Such limitation forbids nesting CIF-inside-a-CIF, but is effectively averted using line prefixing protocol [37], non-standard in CIF 1.1, albeit included in the recent CIF 2 specification [22];
- line lengths typically have to follow a number of recommendations [38].

Since no standard solution exists for the aforementioned issues of arbitrary data presentation for CIF 1.1, we have devised and implemented a protocol to encode and decode file contents prior to their storage in CIF text fields. We implemented a few encoding schemes because of their different, non-overlapping advantages:

- *Numeric Character Reference* (NCR): used in *cod-tools* package [38] to escape binary content and semicolons at the start of lines. This method retains the readability of the text with sparse non-ASCII symbols;
- *Quoted-Printable* [39]: same properties as NCR with an addition the ability to fold long lines;
- *Base64* [39]: overcomes all the deficiencies of CIF at the price of readability and file size; chosen only when the file content is purely binary;
- *gzip+Base64* [39, 40]: same as Base64 with additional file compression.

As one may notice, the gzip+Base64 encoding is composite: it defines a stack of two encodings—Base64-encoding of gzipped contents. To accommodate this and possibly other composite encodings, we define a set of _tcod_content_encoding_* data items, allowing any complex stack of encodings to be described in a CIF loop.

The choice of the encoding is arbitrary and dependent on the requirements of readability and file size expected by the user. Nevertheless, to be able to automate the process of TCOD CIF file generation, we have implemented an algorithm to automatically choose the most appropriate encoding while trying to preserve maximum data readability, as described in “Implementation: exporting the data to the TCOD” section.

To ensure the integrity of both plain and encoded files, checksums are recorded alongside file contents. As of AiiDA v0.10.0, both MD5 and SHA1 algorithms are used.

### Software versioning

While problems of input data versioning may be avoided with revision control systems, WORM (Write Once Read Many) databases, permanent links and checksums, keeping a proper description of data transformations remains a challenging task. In particular, the algorithm of each transformation should be specified in a strictly-defined, machine-readable form. In addition, it would be extremely useful, if not essential, to be able to assert if two different representations will provide the same output when the same input is provided. Basic informatics principles, however, pose a limit on the applicability of such a description, since in general there exists no universal algorithm that could establish the equivalence of two Turing-complete language programs by formally analysing them [41, 42]. In practice, however, algorithms in normalised form could be recorded and claimed to perform identical transformations if their normalised forms are identical. This is hard to achieve, however, since these “descriptions” must be expanded to include compilation/interpretation tools and environment, runtime operating system (OS) and external dynamically-linked libraries. Ideally these parameters should be collected recursively for every dependency. The CPU introduces a final caveat, since two different CPUs, in particular if one or both of them are buggy, can interpret the same algorithm in different ways yielding different results [43]. Thus, in addition to provenance, “descriptions” may be indispensable for an efficient bug tracking. As of AiiDA v0.10.0, data transformations are described by the internal location of executables, runtime command line parameters, environment, execution time stamp and scheduler directives. Moreover, codes from atomistic simulation packages, such as *Quantum ESPRESSO* [44] and *NWChem* [45], which are interfaced with AiiDA, can be queried for version, compilation and runtime parameters. In addition to this, manipulations in the native *Python* environment (referred in AiiDA as “workfunctions”, or “inline calculations”), are also supplemented with the source code. In our experience, this representation is sufficient at the moment and can be easily extended in the future, for instance using virtual machines (e.g. QEMU [46], VirtualBox [47], VMware [48] or similar techniques), or Docker [49], that has rapidly become a widespread tool to reproduce a given computational environment. The presence of full process provenance information in machine-readable format would enable such reconstructions to be performed automatically.

### Bibliographic references

Bibliographic references often appear in CIF files as identifiers of applied algorithms and parameters. A mechanism to provide references in a machine-readable way is described in the cif_core.dic dictionary. According to the recommendations of the International Union of Crystallography (IUCr), citation details should be given in structured tables (CIF loops). In order to categorise references according to the described aspect of the computation (force field, software code etc.), we introduce a data item _tcod_citation_linkage with enumeration values of force-field, software-code, model, pseudopotential, XC-functional and basis-set, to facilitate automatic classification and filtering of computational details based on these attributes. More detailed human-readable description of the relevance can be supplied using _citation_special_details data item.

**Table 1:**
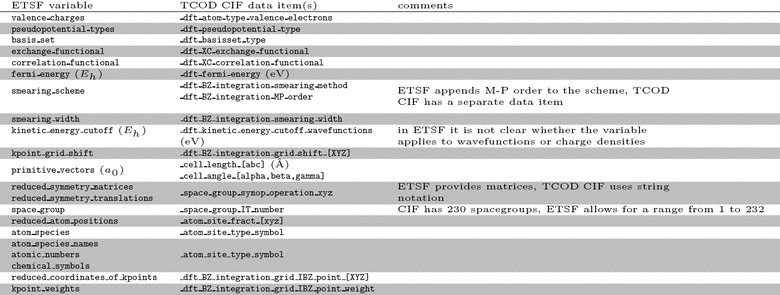
Comparison of a selection of TCOD CIF data items with respect to the corresponding ETSF variables

**Table 2:**
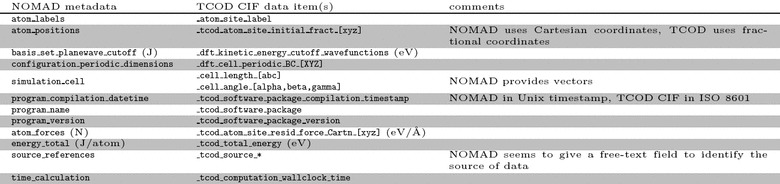
Comparison of a selection of TCOD CIF data items with respect to the corresponding NOMAD metadata

## Ontologies for DFT

The CIF dictionary cif_dft.dic, developed by the advisory board of the TCOD, provides data items for the description of basis sets, pseudopotentials, atomic settings and exchange-correlation functionals. To accommodate input parameters and calculation results exported from AiiDA, we supplement the cif_dft.dic dictionary with data items for Brillouin zone, kinetic energy cut-offs and calculated structure properties, such as the total energy. We also add bulk modulus and stiffness tensor, the latter being represented as a symmetric matrix of 21 independent variables. For the sake of simplicity and consistency with common practices in the core CIF dictionary, we follow the cif_core.dic’s approach to represent matrices in separate “plain” data items (for example, standard anisotropic atomic displacement components in cif_core.dic’s _atom_site_aniso_U_* data items) instead of relational database-style loops, used e.g. by the MPOD [21]. Figure 3 shows a sample from a TCOD CIF file, containing some newly introduced CIF data items.

The ontology defined in the cif_dft.dic dictionary is supplemented by the one that we adopt in this work. For completeness, we mention here that other projects have also invested effort in standardising ontologies for computational materials science simulations, especially for atomistic and/or DFT-based methods. In particular, we report a comparison of the ontology in TCOD CIF with those defined by two other projects, ETSF and NOMAD, in Tables 1 and 2, respectively. Differences are mainly in notations and conventions: for instance, ETSF uses hartree and bohr as main measurement units, NOMAD uses joule and metre, whereas the TCOD uses electronvolt and angstrom. As another example, TCOD choice of using lengths and angles of basis vectors stems from experimental crystallography, while ETSF and NOMAD use vector notation, more common in theoretical materials science. Nevertheless, automatic conversion between the two formats would be easy to implement, making it possible to seamlessly share data between different projects. The TCOD is actively working in contact with other projects to ensure the possibility of such conversion.

## Implementation: exporting the data to the TCOD

The main outcome of this work is the definition and implementation of procedures to export the results of theoretical computations managed with AiiDA into CIF files and deposit them into the TCOD database. To achieve this, we have implemented a converter that, starting from a user-specified structure within the AiiDA database, is able to create a CIF format file. This converter allows for complete automatic *a posteriori* tagging of structures with their metadata. This is made possible by analysing the full provenance (stored in the AiiDA DAG) of the final crystal structure, extracting/converting all relevant information, and storing it in the appropriate CIF fields defined in the TCOD dictionaries discussed before. The generation of CIF files is obtained by interfacing AiiDA with the *cod-tools* package [38]. We summarise here the steps of the export and deposition procedure:

1. *Conversion of periodic structure from internal AiiDA representation to CIF* As of AiiDA v0.10.0, there are two types of representations of periodic structures in AiiDA: structure and trajectory. A structure can be straightforwardly represented in CIF, whereas separate steps of a trajectory can be converted into structures. One or both of these conversions are used to produce an initial template CIF file (containing cif_core.dic data items only), which is supplemented by additional data in the following steps.
2. *Detection of the symmetry and reduction of the unit cell* In AiiDA, modelled materials are represented as non-reduced unit cells of a crystal, in other words, as if their symmetry space groups were *P 1*. Such structures have to be reduced to an asymmetric unit (if possible), leaving out the symmetrically equivalent atoms. To accomplish it, we have harnessed the algorithm by Grosse-Kunstleve and Adams [50], using the implementation in *spglib* [51].
3. *Addition of structure properties (total energies, residual forces etc.)* As much data as possible is parsed from the output of a computation and added to the CIF data items defined by cif_tcod.dic and cif_dft.dic dictionaries, including energy terms and convergence criteria. We have developed a layer to convert the output parameters parsed from the computation outputs by AiiDA into data items of TCOD CIF dictionaries. Currently, the conversion is implemented for both the pw.x and cp.x codes of *Quantum ESPRESSO*, as well as for the *NWChem* package, but the converter has been designed with a modular interface and it can thus be easily extended for any other code interfaced with AiiDA.
4. *Addition of the metadata for reproduction of the results* Since all metadata required for the reproduction of computations (files, scripts, command line strings, etc.) are stored by AiiDA, they are easily collected and stored within the CIF file, along with the description of each AiiDA node used in the exported workflow. Files, consisting of more than a quarter non-ASCII symbols, are assumed to be binary and encoded with Base64, whereas other files with fewer non-ASCII symbols, very long lines or other features, that could cause CIF parsing errors, are encoded with Quoted-Printable. Files larger than one kilobyte (a default value) are gzipped, if requested by the user. There is an option to exclude the contents of files that could be downloaded from the Web via provided URIs thus reducing the size of the resulting CIF file. Checksums are recorded in every case to ensure the integrity of files.
5. *Deposition of generated CIF to the TCOD* The final step is the upload and deposition of the CIF file in the TCOD using the HTTP protocol implemented by the TCOD. The deposition is initiated as an AiiDA calculation, wrapping the generic command line script cif_cod_deposit that is part of the *cod-tools* package. cif_cod_deposit calls the deposition API of the TCOD and transfers the final CIF to the server for validation and deposition, if all checks are passed. The deposition step is optional, so that the final CIF can be simply exported as a local file on disk without deposition. This is useful for instance to manually inspect it before deposition, or if there is a need to share it privately.

With AiiDA installed, CIF generation can be achieved by running, on the command line, the command verdi data structure export --format tcod PK, where PK is the identifier of the structure to export. A large number of command line options exist to customise the behaviour, as explained in the AiiDA documentation. CIF deposition can be achieved instead with the command verdi data structure deposit.

## Discussion

As of October 2017, the number of records in the TCOD has grown to more than 2600. As a proof of concept, over 170 theoretical structures have been deposited to TCOD together with their provenance records using the novel AiiDA–TCOD interface presented here, constituting around 7% of current records in the TCOD. These depositions contain values of total energy in addition to more than 4600 unique AiiDA nodes, that are ready to be automatically imported into user-side AiiDA databases.

To ensure the completeness and semantic integrity of CIF files deposited to the TCOD, automatic checks are performed before accepting contributions. In fact, since CIF dictionaries contain formal descriptions of data items and their values, they can be used for automatic validation of CIF files [52]. A number of tools for automatic CIF data validation already exist, for example IUCr’s checkCIF [53], *iotbx*’s cif.validate [54] and *cod-tools*’s cif_validate. The latter is developed by the COD and TCOD development team and is the one used to validate files upon deposition. In particular, as a part of this work, additional checks have been added to cif_cod_check, the script (part of *cod-tools*) responsible for checking the semantic correctness of deposited data and for its quality control. Checks for the new data items added to the theoretical dictionaries and verification that interrelated data items (data items for coordinates; components of integration grid densities, shifts and residual forces) are simultaneously present, when expected.

Furthermore, in “File content inclusion in CIF and encoding” section we have introduced a number of encodings for files that need to be included within CIF 1.1 files. While the algorithms for decoding these files are known and available on the Web, it is cumbersome for a generic user to implement them in order to decode and extract the files embedded in TCOD CIFs. To address this issue, we have thus developed a program, cif_tcod_tree, to restore the full directory tree used for the execution of the simulation in AiiDA, stored in the TCOD CIF file as described in “Provenance model and directed acyclic graphs” section. After unpacking, the script further fetches remote files that are not embedded in CIF using supplied URIs. Finally, checksums are tested to ensure integrity of the files. The program is available for the end-users as part of *cod-tools*, and can be used to reproduce simulation results seamlessly.

To validate this feature we have asked a collaborator to reproduce the calculations of TCOD entry 10000002 using cif_tcod_tree. As a result of the procedure described here, the collaborator has been able to reproduce the results with great accuracy. The only differences concern the execution timings of *Quantum ESPRESSO*, which are inherently hardware-dependent, thus proving the robustness of our approach.

Provided that software dependencies are met, the workflow can be re-executed running each of its steps in the sequence specified in the CIF file. Finally, output results can be easily compared with the original values provided in the CIF. This makes it possible to run unsupervised replication of deposited results to automatically assess the validity of incoming data. We mention here, however, two aspects that need to be kept in mind when implementing such a service. First, running again the workflows could require a significant amount of computational time (and, for some systems or properties, it is possible to run the workflows only on large clusters). Moreover, particular care has to be taken to prevent damage, accidental or deliberate, of the system replicating the workflow, as well as illegal actions from the network. Runs should be carried out only on isolated or limited systems (i.e., software jails, virtual machines or Docker [49] images). For these reasons, fully-automated workflow replication is not yet implemented in the TCOD. However, we foresee that validation of atomistic simulations could be carried out “on the cloud” in a way very similar to continuous integration services, even harnessing existing tools and infrastructures such as Buildbot [55], Jenkins [56] or Travis-CI [57].

Finally, another important component that we have added to AiiDA, as already discussed in “Input data” section, is the set of importers from structure databases. These (and in particular those for the COD and ICSD) have been already exploited as components of workflows for materials science high-throughput investigations. As an example, in [58] the authors scanned both databases to discover, extract and screen 2D layered structures.

We expect that these tools are going to be even more useful for the computational community in the future, as they are distributed as part of the open-source software AiiDA and therefore freely available as well as extendable. As of now, users of wannier90 [59], VASP [60] and cp2k [61] simulation packages (to name a few, the full list of plug-ins is given in [62]) are able to run calculations as part of AiiDA workflows thanks to community-developed plug-ins. We anticipate that with the growing requirements for reproducibility, and with additional plug-ins for the most popular computational material science software packages, AiiDA and the TCOD will become a convenient solution for the replication problem.

## Conclusions

In this article we have shown the integration of the AiiDA platform (to automatically run and manage scientific workflows while keeping full provenance of the computed data) and of the TCOD (storing computed data associated to crystal structures using an unambiguous ontology, within an open database to facilitate dissemination). Our integration makes it possible to obtain automatic *a posteriori* tagging of crystal structures with metadata, like computed properties and their full provenance (codes adopted, inputs used in the computation, etc.). We have first extended the TCOD CIF dictionaries for atomistic simulations, cif_tcod.dic and cif_dft.dic, to include provenance information. We have then devised means to bypass the intrinsic limitations of the CIF 1.1 file format adopted by the TCOD. Moreover, we have implemented provenance-aware importers into AiiDA from a number of external databases for crystal structures and pseudopotentials. The main outcome of this work is the combination of all these efforts and the implementation of a converter to automatically analyse the data provenance stored in AiiDA after workflow execution, export the results into a CIF file compliant with the TCOD dictionaries, and automatically deposit it into the TCOD. Additionally, we have developed a set of tools for formal quality control and extraction of workflows from CIF files.

The general methodology described in this work does not have to be limited to the TCOD and AiiDA. It may also be implemented in other databases and frameworks, such as GNU Makefile-based replication-ready systems that have been around since as early as 1990 [63]. Our implementation proves that an automation platform to manage simulations and automatically store the full provenance of computed datasets allows metadata to be added at a later time, in a completely automated fashion. Our integration of the TCOD with AiiDA constitutes a fully-open platform implementing all four FAIR principles of “Findability, Accessibility, Interoperability, Reusability” for scientific data management and stewardness [64], that is furthermore fully interlinked with data generation. Indeed, our work allows the deposition in an automated fashion of computational workflows in an open database with permanent URIs and publicly-accessible metadata/dictionaries (*Findability*), that can be, for instance, provided as supplementary material of computational papers. Data is available over standard protocols like HTTP (*Accessibility*) and, thanks to the adoption of the established CIF format and its dictionaries, both data and metadata are fully interlinked (*Interoperability*). Finally, data from the TCOD database together with its full provenance can be easily retrieved and imported back into AiiDA as input for further calculations and analyses (*Reusability*). We expect, therefore, that in the future more researchers will adopt the methods and tools described here to make the data public (as currently required by many funding agencies) with minimal required effort.
